# Current Advances in Comprehending Dynamics of Regenerating Axons and Axon–Glia Interactions after Peripheral Nerve Injury in Zebrafish

**DOI:** 10.3390/ijms22052484

**Published:** 2021-03-02

**Authors:** David Gonzalez, Miguel L. Allende

**Affiliations:** FONDAP Center for Genome Regulation, Facultad de Ciencias, Universidad de Chile, Santiago RM 7800003, Chile; mallende@uchile.cl

**Keywords:** peripheral nerve injury, axonal degeneration, axonal regeneration, peripheral nervous system, Schwann cells, *Danio rerio*, zebrafish

## Abstract

Following an injury, axons of both the central nervous system (CNS) and peripheral nervous system (PNS) degenerate through a coordinated and genetically conserved mechanism known as Wallerian degeneration (WD). Unlike central axons, severed peripheral axons have a higher capacity to regenerate and reinnervate their original targets, mainly because of the favorable environment that they inhabit and the presence of different cell types. Even though many aspects of regeneration in peripheral nerves have been studied, there is still a lack of understanding regarding the dynamics of axonal degeneration and regeneration, mostly due to the inherent limitations of most animal models. In this scenario, the use of zebrafish (*Danio rerio*) larvae combined with time-lapse microscopy currently offers a unique experimental opportunity to monitor the dynamics of the regenerative process in the PNS in vivo. This review summarizes the current knowledge and advances made in understanding the dynamics of the regenerative process of PNS axons. By using different tools available in zebrafish such as electroablation of the posterior lateral line nerve (pLLn), and laser-mediated transection of motor and sensory axons followed by time-lapse microscopy, researchers are beginning to unravel the complexity of the spatiotemporal interactions among different cell types during the regenerative process. Thus, understanding the cellular and molecular mechanisms underlying the degeneration and regeneration of peripheral nerves will open new avenues in the treatment of acute nerve trauma or chronic conditions such as neurodegenerative diseases.

## 1. Introduction

Unlike axons in the central nervous system (CNS), peripheral axons can regenerate and regrow towards their original targets in a well-orchestrated process that involves several steps and players. Following an injury, the axons from the distal stump degenerate through a mechanism known as Wallerian degeneration (WD) [1,2], which is essential for proximal axons to regrow towards their original targets through an unobstructed and proper distal environment. The molecular mechanism underlying WD was begun to be unraveled after the discovery of the spontaneous mouse mutant *Wld*^S^ [3], which encodes the Wld^S^ chimeric protein that slows down distal axonal fragmentation [4,5,6]. After axonal fragmentation, regrowing axons need to go through a bridge formed between the proximal and distal stumps at the injury site; this bridge is mainly composed of extracellular matrix (ECM) components and inflammatory cells [7,8], and its extension varies depending on the length of the injury. Once the regrowing axons have reached the edge of the bridge, they begin extending towards the remains of the distal stump. The distal portion plays a key role in guiding regrowing axons towards their former targets, due to the presence of tubular structures called bands of Büngner, which serve as roads for regenerating axons to reconnect with their original targets [9].

Different cell types such as macrophages, neutrophils and endothelial cells play crucial roles at the multiple stages of the peripheral nerve repair process [8]. However, among these cells, it has been shown that Schwann cells (SCs) play a crucial role during regeneration [10,11]. SCs not only form the myelin sheath along peripheral nerves [10], but they also participate actively in the repair process and are responsible of forming the bands of Büngner at the distal stump. After an injury, mature SCs transdifferentiate through a c-Jun-dependent mechanism, proliferating and becoming repair cells (also known as Büngner cells) [12]. These cells differ from immature SCs and SC precursors found in embryonic nerves, as they down-regulate the expression of myelin-related proteins such as myelin basic protein (MBP) and myelin-associated glycoprotein (MAG) [13] and start upregulating neuron survival-related genes such as GDNF, BDNF, and NT3 [11,12].

Even though much research has been done to elucidate the mechanism underlying the regenerative capacity of peripheral nerves, many questions remain unsolved mostly because of the limitations of the available animal models and tools. In this scenario, the zebrafish has emerged as an attractive animal model as the organization of its nervous system is similar to that of other vertebrates and it exhibits optic transparency at larval stages, accessible genetic manipulation, and most of the genes involved in the development of the nervous system are conserved. Therefore, in combination with the emergence of experimental models of nerve and axonal injury it represents an excellent alternative to study the axonal regeneration dynamics and cell interactions that govern the regenerative process of peripheral nerves.

## 2. Selection Criteria

The aim of this review is to summarize the current advances made in understanding the cellular and molecular mechanisms underlying the regeneration of the PNS in zebrafish. Therefore, we highlight the use of zebrafish coupled with novel tools such as laser-mediated neurectomy and time-lapse microscopy. In this context, we have examined the literature, selecting articles containing one or more of the following keywords: Peripheral nervous system, peripheral nerve injury, axonal degeneration, axonal regeneration, zebrafish, and *Danio rerio*. On the other hand, we have not considered articles related to CNS regeneration in zebrafish.

## 3. Models for Peripheral Nerve Injury in Zebrafish

### 3.1. Posterior Lateral Line Nerve (pLLn)

The lateral line (LL) is a mechanosensory system that is found in amphibians and fish [14]. Although it has been lost in the tetrapod superclass, the internal structure known as the inner ear has been conserved [15]. As a mechanosensory system, the LL responds to mechanical stimuli from water movements and is involved in different behaviors including prey detection and avoidance, and schooling behavior [16,17]. The LL system comprises multiple individual sensory organs called neuromasts, which are composed of 15–20 hair cells surrounded by supporting and mantle cells forming a ring-like structure. The neuromasts are distributed along the body surface in species-specific patterns [15]. The LL sensory system can be subdivided into two major components: (i) The anterior lateral line (aLL), which includes neuromasts on the head and an aLL ganglion (aLLg) located anterior to the ear, while (ii) the posterior lateral line (pLL) includes neuromasts from the trunk to the caudal fin and a pLL ganglion (pLLg) located posterior to the ear [14,15]. Neuromast hair cells are innervated by peripheral bipolar sensory neurons which extend their central axons towards the hindbrain in a somatotopic manner (also referred to as neurotopic), meaning that neurons that innervate anterior neuromasts project their axons towards ventrolateral areas of the hindbrain, while neurons that innervate posterior neuromasts project their axons towards more dorsal areas [14,18,19].

Since the zebrafish pLL has a long and superficial peripheral nerve, and it possesses the typical cell types present in the PNS, it represents an excellent system for studying the degenerative and regenerative process of peripheral axons in vivo. Novel methodologies such laser-mediated nerve transection coupled to time-lapse confocal microscopy, are allowing researchers to study the degeneration and regeneration process of peripheral nerves in living zebrafish [20,21]. In this regard, it has been shown that the pLLn successfully regenerates after an acute injury triggered by laser axotomy in embryonic zebrafish, as observed in other peripheral nerves [22,23]. Thus, the combination of these techniques and the advantages of zebrafish for temporally extended microscopy in the live animal offer a suitable model for studying the mechanisms underlying the degeneration and regeneration of the pLLn. For instance, it has been shown that SCs may be involved in axon fragmentation observed after laser axotomy, since zebrafish embryos lacking SCs showed fewer axon fragments compared to a control group, although the timing of WD was not affected [23]. Interestingly, regenerating axons in zebrafish embryos lacking SCs grew erratically either ventrally or dorsally from their original path while maintaining the same growth rate of control axons, suggesting that SCs might be involved in guiding regrowing axons rather than affecting their growth rate, in both the degeneration and regeneration processes [23]. The role of glial cell line-derived neurotrophic factor (GDNF) in axonal regeneration has also been studied in pLLn of zebrafish embryos upon laser-mediated axotomy. Authors showed that inhibiting the GDNF signaling pathway did not modify the emergence of primary pLL neurons; nonetheless, it slows down axonal regeneration, indicating that the GDNF signaling pathway might be involved in guiding regenerating axons along distal trails of interneuromast cells [22].

Additionally, the regeneration dynamics of the pLLn upon laser ablation were studied in both adult and aging zebrafish. As reported in larval stages, peripheral axons from adult and aging zebrafish pLLn can readily regenerate and reinnervate their targets; however, the onset of the regeneration process occurs in an age-dependent manner, being much delayed in older zebrafish. More interestingly, axonal regeneration is accelerated after a second neurectomy (caudal to the first one), even in aged zebrafish, when performed one week after the first neurectomy. Nonetheless, this regeneration-promoting effect ceases when the second cut is performed 3 weeks after the first one [24]. These observations suggested that a transiently released factor released after the first nerve cut could be promoting axonal regeneration after a second cut. Even more, the authors showed that this effect is likely to depend on axons rather than on SCs, since the promoting effect persists when distal SCs are ablated [24]. A study performed by Xiao et al. [25] showed that SC proliferation was reduced after laser-mediated axotomy of the pLLn, whereas SC apoptosis was increased. In addition, a higher SC apoptosis rate was observed when the pLLg was ablated. SCs were also found to play a role in axonal regeneration, since ErbB2-lacking zebrafish showed an increase in neuronal loss and regrowth failures in regenerating axons. Furthermore, it was shown that proximal and distal SCs helped regenerating axons to regrow at the injury site and they were needed for re-innervating neuromasts properly, suggesting that SCs ease axonal guiding and regeneration, even though they seem not to be essential in the regenerative process after injury [25,26], in concordance with what was previously published [24,27]. Injuries of the adult zebrafish pLLn have also been performed by creating surgical incisions. Using this approach, it has been shown that pLLn regeneration was improved when adult zebrafish were treated with PTP nanofibers, which are aligned polyvinyl cinnamate (PVCi) nanofibers that possess a neural-specific peptide decorated surface and continuously release triiodothyronine (T_3_). PTP nanofiber-treated adult zebrafish also performed better in touch-evoked response test compared to control group, indicating that PTP nanofibers might be improving axonal regeneration and stimulus response by serving as a bridge at the injury site and therefore, facilitating guidance to the regenerating axons [28].

Another injury method called electroablation has been developed [29] and has been found to be suitable for studying tissue regeneration, inflammation, as well as monitoring peripheral nerve regeneration. It produces a localized injury generated by fine microelectrodes and can be used for axotomy of pLL nerves of both embryonic and adult zebrafish. Experiments using this method have shown that the dynamics of the pLLn regeneration is completed by around 25 h post-injury (hpi), similar to what has been reported using two-photon laser axotomy [23]. Using the same approach, it has been shown that reinnervation of neuromasts after neurectomy occurs in a variable fashion, meaning that sensory neurons switch their target neuromast after regeneration and only around one half of sensory neurons reinnervate their original neuromast; however, global somatotopy seems to be preserved after regeneration of pLL axons [27]. A difference in the behavior of proximal and distal SCs was observed, where distal SCs showed increased motility compared to proximal ones and, after 11 hpi, the pLLn fully bridged the ablation gap, confirming previous studies showing an intimate relationship between regenerating axons and SCs. Furthermore, it was shown that SCs were required for the pLLn to regenerate correctly, as the pLLn from zebrafish lacking SCs failed to completely regrow [27]. Interestingly, SCs were also found to be mediators of the neuromast regenerative capacity, since it was observed that they interacted with interneuromast cells during neuromast regeneration. In this context, mature SCs impede proper regeneration by inhibiting interneuromast cells after injury via the ErbB signaling pathway. Thus, the appearance of new neuromasts depends on the interaction between interneuromast cells and SCs in both larvae and adult zebrafish [30], in concordance with a previous study that showed that these two cell types interact during pLL development [31]. Recently, another study has shown that systemic loss of Sarm1, a protein involved in triggering WD, delays axonal degeneration in the zebrafish pLLn, without affecting macrophage infiltration. This study has also shown that macrophage infiltration after injury does not require SCs, since *erbb2^−/−^* zebrafish showed normal macrophage recruitment at the injury site and moreover, Sarm1-lacking axons did not require SCs for their maintenance [32]. These authors also showed that the distal SCs did not transdifferentiate in *sarm1* mutants after injury. It has been previously described that SCs are sensitive to chemotoxicity after nerve injury [33]. In this regard, the authors also found that zebrafish lacking Sarm1 protect SCs from chemotoxicity. Thus, the authors propose that inhibiting Sarm1 might be a possible therapeutic target for treating chronic damage of neurotraumas [32].

**Figure 1:**
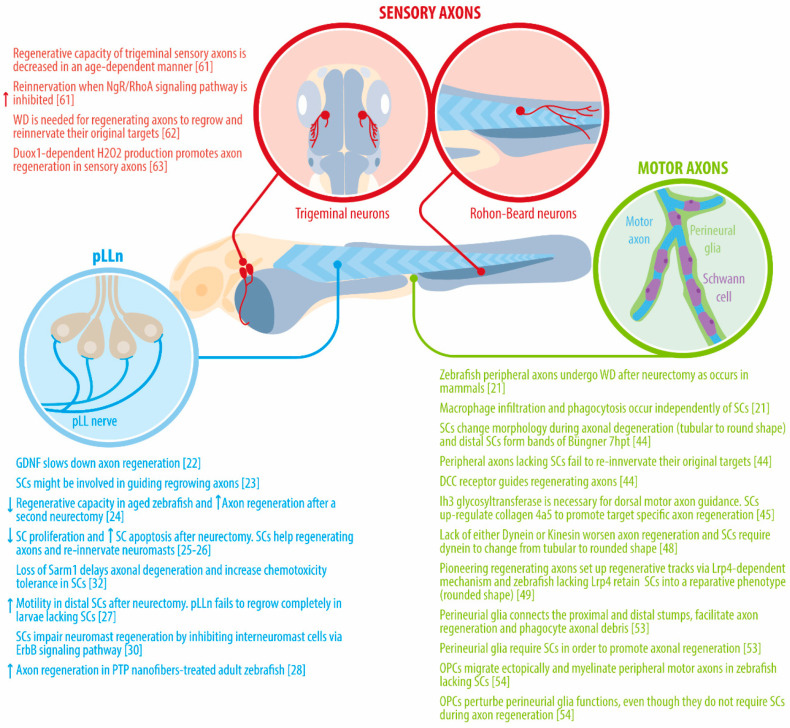
Principal findings on dynamics of axonal regeneration and axon-glia interactions in zebrafish. Thus far, three different groups of peripheral axons have been used to study axon regeneration in zebrafish: the pLLn (blue), which innervates neuromasts along the trunk and tail [22,23,24,25,26,27,28,30,32]; motor axons (green), which elongate their branches towards different areas of the myotomes [21,44,45,48,49,53,54]; and sensory axons (red), specifically trigeminal and Rohon-Beard neurons that innervate the skin of the head and the body [61,62,63], respectively. Dynamics of axonal regeneration have been studied in both embryonic and adult zebrafish by using different experimental approaches such as laser-mediated neurectomy, electroablation of pLLn and surgical incisions coupled to time-lapse microscopy.

Together, these studies provide evidence on the dynamics of degeneration and regeneration of the zebrafish pLLn and the role of different neighboring cells during the regenerative response (Figure 1). Importantly, these processes can be monitored by time-lapse microscopy in both larvae and adult living zebrafish after nerve injury methods such as laser-mediated axotomy, electroablation and surgical incisions. Thus, the zebrafish pLLn represents an excellent model to explore the mechanisms underlying the regeneration of peripheral nerves.

### 3.2. Motor Axons

In zebrafish, motor neurons are classified into two subtypes depending on when they appear and their innervation patterns. Primary motor neurons (PMNs) are the first MNs to appear and can be identified as caudal primary motor neurons (CaP), which are caudally located in the spinal hemisegments and innervate myotomes of the ventral trunk. Rostral primary motor neurons (RoP) are the most rostrally PMNs and innervate myofibers located in the middle area of myotomes, and middle primary motor neurons (MiP) are located in between CaP and RoP PMNs and innervate myofibers from the dorsal myotomes [34,35,36]. Furthermore, variable PMNs (VaP), a fourth subtype of PMNs, are present only in some of the trunk hemisegments and usually die during development around 36 hpf [37]. On the other hand, secondary motor neurons (SMNs) have smaller somata, are located more ventrally in the spinal cord [38] and can be classified into several subtypes based on their innervation pattern as following: Dorsoventrally projecting (dvS), ventrally projecting (vS), and dorsally projecting (dS) SMNs [39]. Thus, they cover the full extent of each muscle segment. Additionally, a second class of SMNs, named intermyotomal secondaries (iS), extend their axons along the intermyotomal area and can be subdivided into iS with collateral projections (iS-c), and iS with no collateral projections (iS-nc) [39].

Since zebrafish motor axons can be easily labeled and monitored in embryonic and adult stages, the dynamics of axonal degeneration and regeneration can be studied in vivo. In this regard, laser-mediated injury was first used in zebrafish with the purpose of ablating motor neurons to further comprehend their role in the migration of neural crest cells [40]. More recently, UV-mediated injury has also been used to damage and induce death of motor neurons in zebrafish, a useful method for studying cellular processes triggered in degenerating neurons [41,42,43].

The pioneering work of Rosenberg et al. [21] combined laser-mediated transection and time-lapse microscopy in living zebrafish and revealed that peripheral motor axons also underwent WD after nerve injury, similar to what has been previously shown in other species. Interestingly, axonal degeneration observed in motor axons occurred in a stereotyped fashion, where almost every peripheral nerve started to fragment between 151 and 210 min after injury, while the distal motor axons from a transgenic line expressing Wld^S^-GFP remained intact up to 8 days after laser-mediated transection, confirming that after transection motor axons degenerate through a Wld^S^-dependent mechanism. Moreover, motor axons were not only able to regrow and re-extend their branches, but injured zebrafish were also able to recover their locomotor activity. Surprisingly, using a *sox10^−/−^* zebrafish line (lacking SCs), the authors showed that macrophage infiltration and phagocytosis after nerve transection occurred independently of the presence of SCs. In further studies, the same group [44] found that SC morphology changed from a tubular shape to a more rounded shape during the first hours of axonal fragmentation, while distal SCs were already forming bands of Büngner by 7 h post-transection (hpt). By 24 hpt, regenerating axons had already crossed the injury site and had reelongated towards their original targets marked by distal SCs. Lastly, regenerating axons fully reestablished their architecture and reinnervated their targets by 48 hpt, as well as SCs recovered their original morphology. Importantly, laser transections performed in motor axons from zebrafish lacking SCs (*sox10^−/−^*; *erbb2^−/−^*; *erbb3^−/−^;* and *nrgltypeIII^−/−^* mutant lines) failed to regrow to their original targets 48 hpt, in concordance with what has been observed in the pLLn [23,27]. Furthermore, the authors found that deleted in colorectal carcinoma (DCC) guidance receptor (a receptor for Netrin) was responsible for axonal guidance during regeneration, confirming for the first time its role in vivo. Another striking study from the Granato laboratory [45] found that the lysyl hydroxylase 3 (Ih3) glycosyltransferase, an enzyme which modifies different types of collagens, was necessary for regenerating dorsal motor axons to regrow and guide them to their original targets. However, this Ih3-dependent guidance was not observed in ventral motor nerves, indicating that successful regrowth of regenerating motor axons depends on specific cues, which selectively lead either dorsal or ventral nerves towards their original paths. Moreover, the authors found that *collagen4a5* mRNA, a substrate for Ih3, was upregulated specifically in a subpopulation of SCs after nerve transection and was responsible of promoting a target-specific regeneration by destabilizing dorsal motor axons that are regrowing aberrantly; and therefore, guiding them towards their original paths (reviewed in [46]). The regenerative capacity of motor axons has also been perturbed after inhibition of Aurora kinase B (AurkB), a serine/threonine kinase mostly studied in different types of human cancers. Although AurkB seems to be promoting axonal regeneration after nerve transection, the underlying mechanisms have not been explored yet. Nevertheless, given that AurkB is involved in modulating microtubule dynamics during cell division, the authors propose that AurkB might be promoting axonal regeneration through a Kif2A-dependent mechanism [47]. On this subject, another work [48] has addressed the role of molecular motors such as kinesin and dynein during axonal regeneration of peripheral nerves in zebrafish larvae. Motor axons from zebrafish lines lacking either dynein (*dync1h1^−/−^*) or kinesin (*kif5aa^−/−^*) failed to regrow properly, while a stronger phenotype was observed in zebrafish lacking dynein. Importantly, motor axons from both *dync1h1^−/−^* and *kif5aa^−/−^* zebrafish larvae showed normal morphology before transection, suggesting that a maternal rescue might be compensating the lack of these proteins during development. Moreover, it was found that SCs require dynein in order to change their morphology from a tubular-like shape into a rounded reparative phenotype, which has been previously described in zebrafish motor axons [44]. Importantly, a further experiment showed that dynein-expressing neurons were able to regrow properly in a *dync1h1^−/−^* zebrafish line, indicating that the neuronal dynein is sufficient to enhance axonal regeneration after nerve transection. Finally, the authors found that dynein promotes early axonal regeneration by stabilizing microtubules to further stabilize their overall regrowth [48]. Using the same experimental approach in zebrafish, it has been demonstrated for the first time that the pioneering regenerating axons set up regenerative tracks for follower regenerating axons to readily regrow via a Lrp4-dependent mechanism. Thus, follower axons regrow faster as they fasciculate with a pioneering axon that has already crossed the injury site. Interestingly, Lrp4 also promotes SC re-differentiation to a myelinating phenotype, since *lrp4^−/−^* zebrafish retained SCs into a reparative phenotype (rounded shape) rather than a tubular-like morphology observed after axons are fully regenerated [49]. Therefore, pioneering and follower axons possess a differential sensitivity to Lpr4 in a regenerative context, indicating that these two subpopulations of regrowing axons have different mechanisms that guide them towards their original targets.

Perineurial glia play a key role during peripheral nerve development [50,51]. However, their role during peripheral nerve regeneration is starting to become unraveled. Using laser-mediated transection [52] a study from 2014 [53] from the Kucenas laboratory showed for the first time in vivo that, at 3 hpn, perineurial glia crosses the injury site forming a bridge that connects the proximal and distal stumps. Even more, perineurial glia remain along the distal nerve even after the clearance of the fragmented distal axons that underwent WD and they facilitate regrowth of regenerating axons. Perineurial glia also form intracellular phagocytic vesicles containing axonal debris and help SCs and macrophages in a spatially coordinated fashion, during the clearance process following axonal degeneration. Lastly, the authors showed that perineurial glia require SCs to promote axonal regeneration, demonstrating that an intimate interaction between these two cell types is necessary for regenerating axons to regrow during the regenerative process. Additionally, another study has showed that in the *erbb3b^−/−^* zebrafish lacking SCs, the oligodendrocyte progenitor cells (OPCs) from the CNS exited the spinal cord and ectopically migrated and myelinated peripheral motor nerves. The authors also found that, when OPCs were ectopically located in caudal motor axons, the perineurial glia were no longer capable of maintaining the bridge at the distal stump. Given that the authors did not find OPCs in the rostral branch of motor axons, they evaluated the perineurial glia behavior in that context and found that they were still able to phagocytose debris and form the bridge in absence of SCs, indicating that even though perineurial glia behavior is perturbed in the presence of OPCs, they do not require SCs for their already mentioned functions during peripheral nerve regeneration [54].

The zebrafish not only represents a suitable model for studying cellular processes in vivo, but it also offers the possibility of doing high-throughput screening to identify small compounds that could be modulating a given biological process in disease context [55,56]. Recently, this approach has been used to identify candidate compounds that modulate peripheral nerve regeneration [57]. In this study, the authors found that peripheral nerves innervating pectoral fin of zebrafish larvae completely regenerated 24 h after fin amputation, and required SCs and FGF signaling as was already demonstrated in mammals [58,59,60]. By using this novel nerve injury assay in zebrafish larvae, they found 21 compounds which hampered axonal regeneration after screening. Moreover, after laser-mediated transection of motor axons [21], the authors found that 6 compounds might be directly involved in perturbing peripheral nerve regeneration in zebrafish larvae: Lavendustin (EGFR inhibitor), dexamethasone (glucocorticoid receptor agonist), AM580 (RA receptor agonist), prostaglandin D2 (PTGDR ligand), verapamil (calcium channel blocker), and 10-HCT (topoisomerase I inhibitor). Thus, a reliable assay has been developed to identify small compounds that could be modulating the regeneration of peripheral nerves [57].

Altogether, the current evidence shows that, as in the zebrafish pLLn, laser-mediated transection of motor axons combined with time-lapse microscopy is starting to shed light on the dynamics of degeneration and regeneration, as well as on the interactions that take place between the regenerating axons and surrounding cell types such as SCs, macrophages and perineural glia, as shown in Figure 1. Importantly, high-throughput screening has identified modulators of axonal regeneration. Therefore, advances in understanding the underlying mechanisms of motor axon regeneration will open new avenues for novel treatments in acute nerve injuries and neurodegenerative diseases.

### 3.3. Sensory Axons

The regeneration of trigeminal sensory axons that innervate the skin has also been studied after two-photon transection in zebrafish [20]. A study from 2009 [61] analyzed the regenerative capacity of peripheral axons at three developmental stages: 30 hpf (trigeminal axons are still growing), 54 hpf (growth of trigeminal axons is completed) and 78 hpf and found that in all stages the distal portion of injured axons degenerated by 3 hpi. However, they found that the capacity for regrowing axons to reinnervate their original targets was significantly decreased in an age-dependent manner. Additionally, it was found that reinnervation was improved when inhibiting Nogo receptor NgR/RhoA signaling pathway at different levels, indicating that this pathway regulates the ability of regrowing axons to reinnervate towards their original targets. A subsequent study by the same group [62] showed that WD of trigeminal axons occurs through three steps: (i) A lag phase, in where distal axons did not show an evident degenerative process, (ii) a rapid and non-progressive phase, in which simultaneous fragmentation take places along the axon and lastly, (iii) a clearance phase, in where all debris is removed. These three phases of WD are distinctly regulated in an age- and axonal length-dependent fashion during zebrafish development, where the lag phase in older zebrafish embryos was significantly faster than younger embryos, while the rapid and clearance phases were the same at the different developmental stages. In addition, the clearance phase rates were found to depend on the axon fragment sizes, suggesting that the clearance phase might be limited by the ability of the different cell types that phagocyte the axonal debris. Furthermore, using a *Wld^S^* zebrafish line, the authors showed that regenerating trigeminal peripheral axons avoided areas in where axon fragments remained, indicating that regrowing axons are repelled by distal axon fragments. Therefore, this study demonstrates that WD is a necessary and coordinated mechanism required for regenerating axons to regrow and correctly reinnervate their original targets. The same group showed in another study [63] that after amputation of zebrafish larvae fins, Rohon-Beard neurons (another subpopulation of sensory neurons that innervate the skin) were capable of reinnervating the tail skin, opposite to the modest regeneration observed in trigeminal neurons after laser-mediated transection [61]. The authors also found that the regenerative capacity of sensory axons was improved when keratinocytes were damaged, and further experiments demonstrated that the regeneration-promoting effect was mediated by Duox1-dependent H_2_O_2_ production [63]. This evidence shows that WD and the regenerative process can be monitored using different populations of peripheral nerves including sensory axons which innervate the skin, a useful model for understanding the role of multiple factors in attracting or repelling regrowing axons to their former targets (Figure 1).

## 4. Concluding Remarks

The versatility of the zebrafish model combined with novel tools is emerging as an excellent approach to study the dynamics of degeneration and regeneration following peripheral nerve injury in vivo. It is also allowing researchers to decipher the spatiotemporal interactions among the different cell types involved in the regenerative process of the PNS, which have been hampered due to the inherent limitations of mammalian models. Pioneering studies performed by O’Brien et al. [20], Schuster et al. [22] and Rosenberg et al. [21] used laser-mediated injury coupled to time-lapse microscopy. Additionally, the use electroablation [27,29] has also allowed researchers to monitor the dynamics of the regenerative process. Importantly, high-throughput screening of small compounds has already identified candidates which can modulate axonal regeneration [57]. Hence, this new perspective will continue expanding our knowledge regarding the cellular and molecular mechanisms underlying peripheral nerve regeneration, and will allow researchers to identify novel molecular targets that modulate the PNS regenerative response and axonal outgrowth; ultimately, leading to new alternatives for treating acute nerve injuries and chronic conditions such as neurodegenerative diseases.
